# Basal Forebrain Volume, but Not Hippocampal Volume, Is a Predictor of Global Cognitive Decline in Patients With Alzheimer's Disease Treated With Cholinesterase Inhibitors

**DOI:** 10.3389/fneur.2018.00642

**Published:** 2018-08-14

**Authors:** Stefan J. Teipel, Enrica Cavedo, Harald Hampel, Michel J. Grothe, Alzheimer's Disease Neuroimaging Initiative, Alzheimer Precision Medicine Initiative (APMI), Lisi Flores Aguilar

**Affiliations:** ^1^German Center for Neurodegenerative Diseases-Rostock/Greifswald, Rostock, Germany; ^2^Department of Psychosomatic Medicine, University of Rostock, Rostock, Germany; ^3^AXA Research Fund and Sorbonne University Chair, Paris, France; ^4^Sorbonne University, GRC n° 21, Alzheimer Precision Medicine, AP-HP, Pitié-Salpêtrière Hospital, Boulevard de l'Hôpital, Paris, France; ^5^Brain and Spine Institute (ICM), INSERM U 1127, CNRS UMR 7225, Boulevard de l'Hôpital, Paris, France; ^6^Department of Neurology, Institute of Memory and Alzheimer's Disease (IM2A), Pitié-Salpêtrière Hospital, AP-HP, Boulevard de l'Hôpital, Paris, France; ^7^IRCCS Istituto Centro San Giovanni di Dio-Fatebenefratelli, Brescia, Italy

**Keywords:** cholinergic treatment, MRI, prediction, memory, executive function, basal forebrain, hippocampus

## Abstract

**Background:** Predicting the progression of cognitive decline in Alzheimer's disease (AD) is important for treatment selection and patient counseling. Structural MRI markers such as hippocampus or basal forebrain volumes might represent useful instruments for the prediction of cognitive decline. The primary objective was to determine the predictive value of hippocampus and basal forebrain volumes for global and domain specific cognitive decline in AD dementia during cholinergic treatment.

**Methods:** We used MRI and cognitive data from 124 patients with the clinical diagnosis of AD dementia, derived from the ADNI-1 cohort, who were on standard of care cholinesterase inhibitor treatment during a follow-up period between 0.4 and 3.1 years. We used linear mixed effects models with cognitive function as outcome to assess the main effects as well as two-way interactions between baseline volumes and time controlling for age, sex, and total intracranial volume. This model accounts for individual variation in follow-up times.

**Results:** Basal forebrain volume, but not hippocampus volume, was a significant predictor of rates of global cognitive decline. Larger volumes were associated with smaller rates of cognitive decline. Left hippocampus volume had a modest association with rates of episodic memory decline. Baseline performance in global cognition and memory was significantly associated with hippocampus and basal forebrain volumes; in addition, basal forebrain volume was associated with baseline performance in executive function.

**Conclusions:** Our findings indicate that in AD dementia patients, basal forebrain volume may be a useful marker to predict subsequent cognitive decline during cholinergic treatment.

## Introduction

A prediction of the individual course of cognitive change in Alzheimer's disease (AD) would help adequate resource allocation, patient care, and counseling. Evidence suggests that the hippocampus supports the consolidation of long-term declarative memory (1, 2), showing neurodegeneration in autopsy data and atrophy in *in vivo* MRI scans as early as in predementia stages of AD (3, 4). Its measurement is standardized, robust, accessible and feasible for *in vivo* studies using established volumetric protocols, with the most recent advance being an internationally harmonized protocol for a consistent delineation of the hippocampus' anatomical borders on MRI scans (5). The cholinergic basal forebrain is the main source of neocortical acetylcholine (6), and is involved in attentional processes, such as immediate recall and executive function (7). Autopsy studies found degeneration of cholinergic basal forebrain neurons in early clinical stages of AD (8, 9), and the resulting reduction of cholinergic cortical activity represents the rationale for the use of cholinergic treatment in AD dementia. The hippocampus represents a key input area of cholinergic projections from the basal forebrain (10). In recent years, MRI based protocols for an automated measurement of cholinergic basal forebrain volumes have been established that make use of stereotactic information derived from combined post mortem MRI and histology (11–13). Based on these protocols, several MRI volumetric studies have shown consistent pattern of hippocampus and cholinergic basal forebrain atrophy in AD dementia (11, 14) and prodromal at-risk stages of AD dementia, such as amnestic mild cognitive impairment (MCI) (15, 16) or individuals with amyloid positive MCI (17).

Based on these findings, hippocampus and cholinergic basal forebrain volumes may help to predict cognitive change and response to cholinergic treatment in patients with AD dementia or prodromal AD. A previous study found that the thickness of the substantia innominata, a potential proxy of cholinergic basal forebrain integrity (18), was associated with rates of cognitive change in 82 AD dementia patients during 9 months of treatment with a cholinesterase inhibitor, with smaller rates of cognitive decline in people with a lower thickness of the substantia innominata (19). In people with MCI, hippocampus volume was associated with rates of cognitive decline with a moderate effect size (20–24). In 37 AD dementia cases, smaller hippocampus volume was associated with faster global cognitive decline during cholinergic treatment (25). In a recent randomized controlled trial of donepezil, we found that hippocampus volume, but not basal forebrain volume, was a predictor of subsequent cognitive decline in 216 MCI cases (26); this effect, however, was independent of treatment. In summary, in MCI cases hippocampus volume, but not basal forebrain volume, was found to be a significant predictor of cognitive decline, irrespective of treatment. In studies on AD dementia cases, hippocampus volume and a proxy for basal forebrain volume were found significant predictors of cognitive decline during cholinergic treatment.

Here, we used longitudinal cognitive data of 124 AD dementia cases retrieved from the Alzheimer's Disease Neuroimaging Initiative (ADNI-1) database, all receiving cholinergic treatment. Based on the previous evidence on the potential predictive value of these brain regions in AD, we determined the association of hippocampus and basal forebrain volume with rates of global and domain-specific cognitive decline during cholinergic treatment. We expected that lower basal forebrain (hippocampus) volume would predict a faster rate of global and executive (global and episodic memory) function. Secondly, we determined the predictive use of basal forebrain and hippocampus volumes for the identification of cognitively stable vs. cognitively declining patients, where we expected that cognitively stable patients would have larger basal forebrain and hippocampus volumes at baseline. These data help to assess the potential usefulness of volumetric MRI to identify people with a more rapid disease progression; such data would support clinical decision making on allocation of treatment resources and care.

## Methods

### Study population

Data used in the preparation of this article were obtained from the Alzheimer's Disease Neuroimaging Initiative (ADNI) database (adni.loni.usc.edu). The ADNI was launched in 2003 as a public-private partnership, led by Principal Investigator Michael W. Weiner, MD. The primary goal of ADNI has been to test whether serial magnetic resonance imaging (MRI), positron emission tomography (PET), other biological markers, and clinical and neuropsychological assessment can be combined to measure the progression of mild cognitive impairment (MCI) and early Alzheimer's disease (AD). A fuller description of ADNI and up-to-date information is available at www.adni-info.org. We retrieved data of participants of the ADNI-1 study who had a clinical diagnosis of AD dementia at baseline, a baseline MRI scan, neuropsychological testing at baseline and follow-up and documented treatment with any cholinesterase inhibitor during follow-up time. We retrieved 124 cases, 56 women, fulfilling these conditions. We included only people with a clinical diagnosis of AD dementia, because cholinesterase inhibitor treatment is only approved for this diagnosis, but not for MCI or other diagnoses.

### Neuropsychological tests

We used ADAScog11 as measure of global cognitive decline (27–29). ADAScog 11 has frequently been used in clinical efficacy trials of cholinesterase inhibitors in AD as primary endpoint (30, 31). In addition, we used composite measures for memory and executive function, respectively, to account for the different versions of the word lists of neuropsychological tests employed in the ADNI psychometric assessment. The ADNI composite scores have been previously defined and they appear to: (i) have good validity, (ii) include additional information, incorporating all of the domain-specific information available from the neuropsychological battery administered in ADNI, and (iii) be strongly associated with a priori specified neuroimaging parameters selected on the basis of their known association with the respective cognitive domain (32, 33).

### MRI acquisition

ADNI MRI data were acquired on multiple 1.5 Tesla MRI scanners using phantom-calibrated scanner-specific T1-weighted sagittal 3D MPRAGE sequences. In order to increase signal uniformity across the multicenter scanner platforms, original MPRAGE acquisitions in ADNI undergo standardized image pre-processing correction steps. Standardization of MRI sequences across ADNI sites and centralized image pre-processing steps have been described in detail previously (34) and are documented on the ADNI website (http://adni.loni.usc.edu/methods/).

### MRI data processing

The processing of structural MRI scans was implemented through statistical parametric mapping, SPM8 (Wellcome Dept. of Imaging Neuroscience, London), and the VBM8-toolbox (http://dbm.neuro.uni-jena.de/vbm/) implemented in MATLAB 7.1 (Mathworks, Natwick), and has been described in detail previously (35, 36). Briefly, MRI scans were automatically segmented into gray matter (GM), white matter (WM), and cerebrospinal fluid (CSF) partitions of 1.5 mm isotropic voxel-size, using the segmentation routine of the VBM8-toolbox. The resulting GM and white matter partitions of each subject in native space were then high-dimensionally registered to an aging/AD-specific reference template based on a completely independent cohort (37) using the Diffeomorphic Anatomic Registration using Exponentiated Lie algebra (DARTEL) algorithm (38). Individual flow-fields obtained from the DARTEL registration to the reference template were used to warp the GM segments and voxel-values were modulated for volumetric changes introduced by the high-dimensional normalization, such that the total amount of GM volume present before warping was preserved. All preprocessed GM maps passed a visual inspection for overall segmentation and registration accuracy.

The total intracranial volume (TIV) was calculated as the sum of the total segmented GM, WM, and CSF volumes (38). GM volumes of the hippocampus and basal forebrain cholinergic nuclei were automatically extracted by summing up the modulated GM voxel values within respective regions of interest (ROI) in the reference space. The basal forebrain ROI was based on a recently published cytoarchitectonic map of basal forebrain cholinergic nuclei in MNI space, derived from combined histology, and in cranio MRI of a post-mortem brain (13). This cytoarchitectonic map matches standard MNI space and was projected into the aging-AD specific template space using non-linear warping parameters obtained from a DARTEL registration. Although the cytoarchitectonic basal forebrain map comprises detailed outlines of different cholinergic subdivisions within the basal forebrain, including cell clusters corresponding to the medial septum, diagonal band, nucleus subputaminalis, and nucleus basalis Meynert, in the current study we only considered the entire volume of the map, including all cholinergic subdivisions, as a proxy for overall basal forebrain cholinergic system integrity. The ROI mask for the hippocampus was obtained by manual delineation of the hippocampus in the reference template of aging-AD specific anatomy (37) using the interactive software package Display (McConnell Brain Imaging Centre at the Montreal Neurological Institute) and a previously described protocol for segmentation of the medial temporal lobe (39). An illustration of both ROIs in the gray matter fraction of the reference space template employed in our current study can be found in the previous publications (15, 40, 41).

### Statistical analysis

We conducted two types of analysis. The first analysis determined associations of volumetric markers with rates of change in cognitive scores as continuous outcomes. The second analysis determined the accuracy of response prediction in cognitive scores as binary outcome.

#### Association with rates of change

We determined the main effects and the two-way interactions of baseline volumes by time on neuropsychological performance as dependent variable using linear mixed effects models with subject-related random effects for intercept and time, controlling for age, sex, and TIV. The model fit was compared between nested models (random intercept vs. uncorrelated random intercept and slope vs. correlated random intercept and slope) using Akaike's information criterion (AIC) (42). Significance of parameters was determined using t-statistics with degrees of freedom determined according to the Satterthwaite approximation. Mixed effect model analyses were calculated in R, including the libraries “lme4” and “lmerTest,” available at http://cran.r-project.org/web/packages.

#### Response prediction

We originally had planned to determine response prediction. Similarly to previous studies (43), we defined response as more than 4 points improvement (i.e., at least 4 points decline) in ADAScog11 over one year. This criterion, however, yielded only three responders so that an analysis was not feasible. Consequently, we relaxed the response criterion and discriminated between non-decliners (zero change or better) vs. decliners in the cognitive endpoints. Rates of change in cognitive scores were derived from the coefficients of the subject-related random effect for time on the cognitive scores, controlling for age and sex. We determined logistic regression models regressing the binary endpoint of decline vs. non-decline on those volumetric markers that had shown a significant association with the continuous rates of cognitive decline in the previous models.

We used block-wise cross validation with repeated random sampling, based on Gaussian-distributed random numbers generated in R. We repeatedly split the data set into 63.2% of training data and 36.8% of test data. For each of the repeatedly drawn training samples, the logistic regression parameters were estimated and subsequently applied to the remaining test data set. Bootstrapping aimed to assess levels of predictive accuracy in the test data so to avoid overestimation of accuracy levels that occurs when assessment is based on the training data. We recorded areas under the receiver operating characteristic curves (AUC) for each test data set; different to the rate of correctly identified cases, the AUC is insensitive to an uneven distribution of outcomes. The entire cross-validation process was iterated 100 times to determine the variability of the estimates of accuracy across runs. We determined nonparametric bootstrap confidence intervals with the 2.5 and 97.5 percentiles defining the lower and upper limits of the confidence interval [(44), Chapter 13]. Logistic regression analysis was calculated in R, using function glm with the parameter family = binary.

All analyses were performed with RStudio, version 0.98.1102, a user interface of R Project for Statistical Computing Analyses.

## Results

### Sample

We retrieved 124 (56 women) cases with AD dementia fulfilling the inclusion criteria. Follow-up times ranged between 0.4 and 3.1 years, number of follow-up time points ranged between 1 and 5. Baseline demographic characteristics and hippocampus and basal forebrain volumes are given in Table 1.

**Table 1:** Baseline demographic characteristics.

N (women)	124 (56)
Age, mean (*SD*) in years	75.3 (7.4)
MMSE, mean (*SD*)	23.5 (1.9)
ADAScog11, mean (*SD*)	19.0 (6.4)
ADNI-MEM, mean (*SD*)	−0.9 (0.5)
ADNI-EXE, mean (*SD*)	−0.9 (0.8)
L. hippocampus volume (*SD*) in mm^3^	2,038 (337)
R. hippocampus volume (*SD*) in mm^3^	2,212 (374)
Basal forebrain volume (*SD*) in mm^3^	481 (85)

*ADAScog11 - 11-items version of the Alzheimer's Disease Assessment Scale–Cognitive subscale, higher values indicate worse performance*.

*ADNI-MEM - ADNI memory score (32); this scale provides z-standardized performance scores, lower values indicate worse performance*.

*ANDI-EXE - ADNI executive function score (33); this scale provides z-standardized performance scores, lower values indicate worse performance*.

### Association with global and domain-specific cognitive rates of change

**Figure 1:**
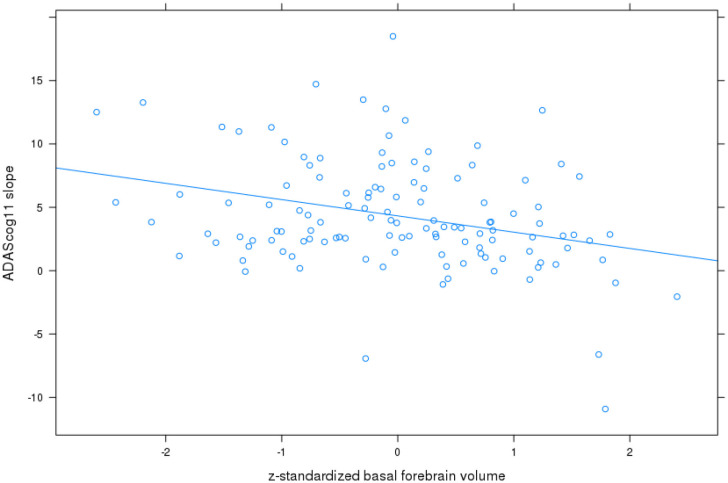
Basal forebrain volume and rates of change in ADAScog11. Plot of z-standardized basal forebrain volume on mixed effects linear model estimates of rates of change in ADAScog score controlling for age and sex, with linear regression line.

For **ADAScog11**, the best fit was achieved with a model allowing for a correlated random intercept and slope. Detailed results are shown in Table 2. ADAScog11 showed a significant worsening over time with 4.3 points increase per year. Left and right hippocampus and basal forebrain volumes were significantly correlated with ADAScog11 baseline performance, with better performance with higher volume. In addition, basal forebrain volume was associated with less worsening in ADAScog11 performance over time (*t* = −2.9, 115 df, *p* < 0.005) with the effect amounting to 1.6 points less increase in ADAScog11 per year when the volume of basal forebrain was one standard deviation higher (Figure 1). The partial correlation coefficient (controlling for TIV) between slopes of ADAScog11 change from the mixed effects model and basal forebrain baseline volume was *r* = −0.23, *p* < 0.01. Left and right hippocampus volumes had no significant effects on the ADAScog11 rates of change over time (*p* > 0.22 for all comparisons).

**Table 2:** Summary of predictor effects.

		**Left Hp t(df); *p***	**Right Hp t(df); *p***	**BF t(df); *p***
**ADAScog11**	Volume	−2.1(120); <0.04	−3.6 (120); <0.001	−3.0(119); <0.004
	Time	8.0(109); 10^−11^	8.0(109); 10^−11^	8.0(109); 10^−11^
	Time*Volume	n.s.	n.s.	−2.9(115); <0.005
**ADNI-MEM**	Volume	2.3(129); <0.03	3.3(128); <0.002	3.9(128); <0.001
	Time	−10.7(112); 10^−14^	−10.7(112); 10^−14^	−10.7(112); 10^−14^
	Time*Volume	−2.0(109); <0.05	n.s.	n.s.
**ADNI-EXE**	Volume	n.s.	n.s.	3.2(123); <0.002
	Time	−10.1(98); 10^−14^	−10.1(98); 10^−14^	−10.1(98); 10^−14^
	Time*Volume	n.s.	n.s.	n.s.

*Reported are the main effect of time and volume and the interaction effects of volume by time. All models were controlled for age, sex, and total intracranial volume*.

*Data are given as: t-values (t); number of degrees of freedom (df); p-value (p)*.

*Please note: the sign of the t-value gives the direction of the effect, i.e., a positive t-value indicates that test scores were higher with higher volume and vice versa*.

*Hp, hippocampus*.

*BF, basal forebrain*.

*ADAScog11, 11-items version of the Alzheimer's Disease Assessment Scale–Cognitive subscale*.

*ADNI-MEM, ADNI memory score (32)*.

*ANDI-EXE, ADNI executive function score (33)*.

**Figure 2:**
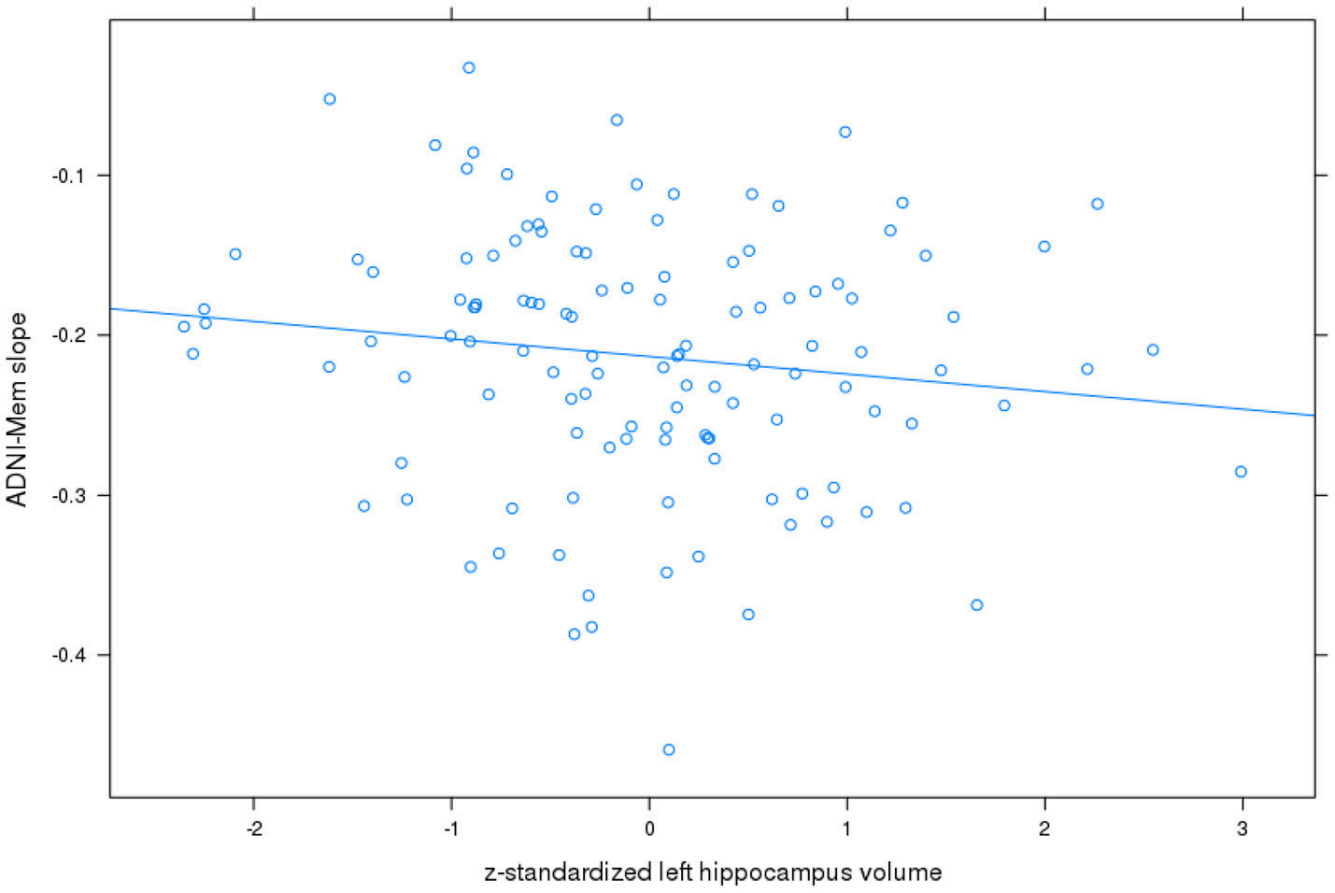
Left hippocampus volume and rates of change in ADNI memory score. Plot of z-standardized left hippocampus volume on mixed effects linear model estimates of rates of change in ADNI memory (ADNI-Mem) score controlling for age and sex, with linear regression line.

For **ADNI memory score**, the best fit was achieved with a model allowing for an uncorrelated random intercept and slope. Detailed results are shown in Table 2. On average, patients lost 0.21 z-score points per year. Left and right hippocampus and basal forebrain volumes were significantly associated with baseline memory performance, with higher performance associated with higher volume. In addition, left hippocampus volume was associated with less worsening in the ADNI memory score over time (*t*= −2.0, 109 df, *p* < 0.05) with the effect amounting to 0.04 less z-score points lost per year when the volume was one standard deviation higher (Figure 2). The partial correlation coefficient (controlling for TIV) between slopes of ADNI memory rates of change from the mixed effects model and left hippocampus baseline volume was −0.15, *p* < 0.1. Right hippocampus and basal forebrain volumes had no significant effects on the ADNI memory rates of change over time (*p* > 0.16 for all comparisons).

For **ADNI executive function score** the best fit was achieved with a model allowing for an uncorrelated random intercept and slope. Detailed results are shown in Table 2. On average, patients lost 0.29 z-score points per year (*t* = −10.1, 98 df, *p* < 0.001). Basal forebrain was significantly associated with baseline executive function performance (*t*= 3.2, 123 df, *p* < 0.002), with better performance associated with higher volume; there was no effect for left or right hippocampus volume. Neither bilateral hippocampus nor basal forebrain volumes had a significant effect on the executive function rates of change over time (*p* > 0.20 for all comparisons).

The covariates that were used in the model showed no effect of age, sex or total intracranial volume on ADAScog; age had a significant effect on ADNI memory and executive function scores (*p* < 0.05), but sex and TIV had no significant effects on these scores.

### Response prediction

Basal forebrain volume was significantly associated with the outcome of non-decline in ADAScog11 with an odds ratio of 2.5 (*p* < 0.05), i.e., a one standard deviation higher basal forebrain volume increased the odds of non-decline by a factor of 2.5. The corresponding bootstrapped AUC in the test data was 0.78, the 2.5/97.5 percentile confidence interval was 0.50 to 0.98. For the ADNI memory score there were no non-decliners, rendering further analysis infeasible.

## Discussion

We found significant decline of global cognitive function as well as memory and executive function in AD dementia cases during follow-up. Higher basal forebrain volume was associated with slower global cognitive decline, and higher left hippocampus volume was associated with slower memory decline. The prognostic use of basal forebrain volume to discriminate between cognitive decliners and cognitive stable persons based on the global ADAScog11 score reached a cross-validated area under the ROC curve of 0.78, indicating a fair accuracy, however with a broad bootstrapped confidence interval including the random guessing level of AUC = 0.5.

The clear decline of global cognitive function as assessed by ADAScog11 is consistent with previous studies on the course of cognitive decline in dementia stages of AD (45). A large cohort study of 622 cases with cholinesterase inhibitor treatment and paired assessments of MMSE at baseline and after 3 to 4 months showed a response rate of 37%, when defining response by at least 2 points MMSE increase (46). The findings in this large cohort study agree with findings in randomized controlled trials with cholinesterase inhibitors showing an increase of MMSE or decrease in ADAScog scores in the first 3 to 6 months of treatment with subsequent decline (47, 48). Here, we assessed longer term follow-up, between 0.4 and 3.1 years, accounting for the low number of cases fulfilling response criteria (only 10 of 124 cases, even when defining response as non-decline).

In the cross-sectional analyses, baseline volumes of basal forebrain, and left and right hippocampus were associated with global cognitive function as well as memory performance, as assessed by the ADNI memory composite score. These findings agree with previous studies showing that hippocampus volume was associated with episodic memory (49) and global cognitive (50) performance in AD patients. Similarly, previous studies have described associations of basal forebrain volume with global cognitive and episodic memory performance in AD dementia and MCI cases in cross-sectional analysis (15, 40, 41). In addition, in the current study, basal forebrain volume was associated with the executive function composite score. This agrees with the observation that cholinergic activity subserves executive function and attention that is supported by the adverse effects of anticholinergic treatment on executive function and attention (51–53), and by findings of similar associations in independent cohorts (15, 40, 41).

Cholinergic basal forebrain volume was significantly associated with subsequent global cognitive decline as assessed by ADAScog11. AD dementia cases with a one standard deviation higher basal forebrain volume had 1.6 points less worsening in ADAScog11 per year, accounting for 37% of the annual overall rate of ADAScog11 worsening. Basal forebrain volume allowed correct discrimination between cognitive non-decliners and cognitive decliners with an AUC of 0.78. We used the AUC as measure of accuracy, because this measure is less sensitive to the proportion of non-decliners; in contrast, the level of correct case identification would have been uninformative, because with only 10 non-decliners simply predicting non-conversion in all cases would already yield correct classification in 114 out of 124 cases. Our findings indicate that in the presence of cholinergic treatment, a high cholinergic basal forebrain volume is associated with more benign global cognitive decline. Our findings agree with a previous exploratory study, where response to cholinergic treatment over 9 months as measured by the MMSE score was significantly associated with gray matter volume in basal forebrain regions from a voxel based regression analysis in 23 AD cases (54). Our findings disagree with a study in 82 AD dementia patients using the substantia innominata thickness as a proxy of cholinergic basal forebrain integrity (18); here smaller rate of cognitive decline was found associated with a smaller thickness of the substantia innominata during 9 months cholinergic treatment (19). The number of subjects was higher and the average follow-up time was longer in our study compared to the previous study. In addition, manual measurement of the thickness of the substantia innominata is prone to intra- and inter-rater variability, and assesses only a small subsection of the cholinergic basal forebrain compared to the automated measurement of basal forebrain volume based on a post mortem reference map (11).

Hippocampus volume was not associated with the subsequent rate of global cognitive decline. In a small sample of 37 AD patients, a previous study found a higher hippocampus volume associated with less worsening of ADAScog score over 0.5 to 2 years of follow-up (25). A part of these 37 individuals had been classified as very mild AD, resembling rather the prodromal MCI than the dementia stage of AD. This outcome therefore agrees with the results of our previous study on 216 MCI cases, where we found that higher hippocampus volume, rather than basal forebrain volume, was associated with more benign rates of global cognitive and memory decline (26). Taken together these findings suggest that hippocampus volume may be a proxy of reserve capacity in MCI individuals, but no more in AD dementia patients. This interpretation would agree with the notion that hippocampus atrophy begins to degenerate earlier than basal forebrain and reaches a plateau in the dementia stage of AD so that the functional relevance of hippocampus volume variation would be limited in the dementia stage of the disease. In contrast, changes in basal forebrain volume may still dynamically progress during AD dementia and may thus serve as a better predictor of disease progression in more advanced disease stages (37).

In contrast to global cognitive decline, left hippocampus volume was significantly associated with the rate of episodic memory score decline, consistent with previous evidence in subjects with MCI (26, 55). Different to global cognitive decline we could not determine the predictive accuracy of hippocampus volume for non-decline, as non-decline did not occur in our sample. The partial r of −0.15, however, points to a small effect size for left hippocampus volume on subsequent rate of memory decline, consistent with a limited role of hippocampus volume for predicting subsequent cognitive decline in the AD dementia stage, even when considering the hippocampus-specific functional measure of episodic memory performance.

Beyond structural markers, such as hippocampus or basal forebrain volumes, a previous study found cortical network functional integrity in functional MRI as a significant predictor of cholinergic treatment response, but the study included only 18 cases (56).

Several limitations have to be considered with our study. First, similar to previous studies in AD dementia (19, 25) we determined the predictive value of hippocampus and basal forebrain volumes for cognitive decline in patients who all received treatment. Therefore, we can only derive conclusions on prediction of cognitive decline during treatment, but not on prediction of treatment effects; in our view this distinction is sometimes not made explicit enough in the literature (19, 25). We had decided to exclude AD dementia cases without documentation of cholinergic treatment since the lack of documentation of treatment may not be a reliable indicator for the lack of treatment in the ADNI cohort. In addition, the lack of treatment in a cohort like ADNI will likely be related to selection bias; the ADNI cohort by design features no random allocation to treatment. Furthermore, information on the duration of treatment before inclusion in the ADNI cohort was not available so that stratification according to duration of treatment was not possible. Our findings encourage the analysis of prospective controlled clinical trials in AD dementia for a potential association of basal forebrain and hippocampus volumes with rates of subsequent cognitive change in dependency of treatment. Secondly, observation periods were very heterogeneous in the ADNI cohort. We used a mixed effects model to explicitly model variability in observation periods. Indeed, the models including a random effects term for time provided a better fit than models excluding such term. Thirdly, it would have been interesting following previous evidence on the corticotopic organization of the cholinergic basal forebrain to analyze a differential involvement of antero-medial vs. postero-lateral basal forebrain subregions. Anterior-medial basal forebrain nuclei project mainly to the hippocampus and ventromedial cortical regions, whereas posterior-lateral nuclei project more densely to lateral neocortical areas (6, 57, 58), which may also be related to different functional representation of these subnuclei. However, the overall small size of the basal forebrain volume restricts the accuracy of subregional assessments so that we did not include such an analysis. Fourthly, we had selected the ADNI memory and executive function composite scores to reduce the dimensionality of our analyses. Previous studies had shown that both composite measures exhibited more consistent rates of change in MCI and AD dementia individuals than the respective single tests (32, 33). Finally, we aimed to determine odds ratios of volumes for predicting clinically significant response to treatment. Such response has previously been defined to equal at least 4 points decrease in ADAScog11 (43). Since only 3 cases fulfilled this response criterion, we could not conduct the intended analysis. When we used a more liberal criterion of no cognitive decline, i.e., ≤ 0 points change in ADAScog11, we found a significant odds ratio of 2.5. This indicates that a person with a one standard deviation higher basal forebrain volume has a 2.5 higher chance of no decline, all other variables kept constant. However, this analysis detects a clinically potentially less relevant endpoint than the originally planned analysis of 4 points change.

In summary, we found significant decline of global cognitive function as well as memory and executive function in AD dementia patients treated with cholinesterase inhibitors. Basal forebrain volume, but not hippocampus volume, was a predictor of global cognitive decline with a cross-validated accuracy of approximately 78% to discriminate between non-decliners and decliners, albeit based on a small sample of non-decliners. In contrast, left hippocampus volume showed only a modest association with subsequent rates of memory decline during cholinergic treatment. Our data suggest that with the transition from prodromal MCI to AD dementia the brain areas with biologically meaningful dynamic variation and ensuing predictive value may shift from the hippocampus to the basal forebrain region. The use of hippocampus and basal forebrain volumes to predict response to cholinergic treatment in AD dementia needs to be studied in cohorts with controlled treatment.

## Ethics approval and consent to participate

All procedures performed in the ADNI studies involving human participants were in accordance with the ethical standards of the institutional research committees and with the 1964 Helsinki declaration and its later amendments. Written informed consent was obtained from all participants or their authorized representatives.

The study procedures were approved by the institutional review boards of all participating centers (https://adni.loni.usc.edu/wp-content/uploads/how_to_apply/ADNI_Acknowledgement_List.pdf:) Oregon Health and Science University; University of Southern California; University of California—San Diego; University of Michigan; Mayo Clinic, Rochester; Baylor College of Medicine; Columbia University Medical Center; Washington University, St. Louis; University of Alabama at Birmingham; Mount Sinai School of Medicine; Rush University Medical Center; Wien Center; Johns Hopkins University; New York University; Duke University Medical Center; University of Pennsylvania; University of Kentucky; University of Pittsburgh; University of Rochester Medical Center; University of California, Irvine; University of Texas Southwestern Medical School; Emory University; University of Kansas, Medical Center; University of California, Los Angeles; Mayo Clinic, Jacksonville; Indiana University; Yale University School of Medicine; McGill University, Montreal-Jewish General Hospital; Sunnybrook Health Sciences, Ontario; U.B.C. Clinic for AD & Related Disorders; Cognitive Neurology—St. Joseph's, Ontario; Cleveland Clinic Lou Ruvo Center for Brain Health; Northwestern University; Premiere Research Inst (Palm Beach Neurology); Georgetown University Medical Center; Brigham and Women's Hospital; Stanford University; Banner Sun Health Research Institute; Boston University; Howard University; Case Western Reserve University; University of California, Davis—Sacramento; Neurological Care of CNY; Parkwood Hospital; University of Wisconsin; University of California, Irvine—BIC; Banner Alzheimer's Institute; Dent Neurologic Institute; Ohio State University; Albany Medical College; Hartford Hospital, Olin Neuropsychiatry Research Center; Dartmouth-Hitchcock Medical Center; Wake Forest University Health Sciences; Rhode Island Hospital; Butler Hospital; UC San Francisco; Medical University South Carolina; St. Joseph's Health Care Nathan Kline Institute; University of Iowa College of Medicine; Cornell University and University of South Florida: USF Health Byrd Alzheimer's Institute.

## Availability of data and materials

The datasets analyzed during the current study are available in the ADNI repository, www.adni-info.org.

## Author contributions

ST and MG have made substantial contributions to conception and design of the study, and the analysis and interpretation of data, drafted the manuscript, given final approval of the version to be published, agreed to be accountable for all aspects of the work in ensuring that questions related to the accuracy or integrity of any part of the work are appropriately investigated and resolved. EC and HH have made substantial contributions to analysis and interpretation of data, been involved in revising the manuscript critically for important intellectual content, given final approval of the version to be published, agreed to be accountable for all aspects of the work in ensuring that questions related to the accuracy or integrity of any part of the work are appropriately investigated and resolved.

### Conflict of interest statement

The authors declare that the research was conducted in the absence of any commercial or financial relationships that could be construed as a potential conflict of interest. The reviewer KS and handling Editor declared their shared affiliation.

## References

[1] Carr VA Viskontas IV Engel SA Knowlton BJ. Neural activity in the hippocampus and perirhinal cortex during encoding is associated with the durability of episodic memory. J Cogn Neurosci. (2010) 22:2652–62. 10.1162/jocn.2009.21381 19925190

[2] Deweer B Pillon B Pochon JB Dubois B. Is the HM story only a “remote memory”? Some facts about hippocampus and memory in humans. Behav Brain Res. (2001) 127:209–24. 10.1016/S0166-4328(01)00366-7 11718893

[3] Price JL Morris JC. Tangles and plaques in nondemented aging and “preclinical” Alzheimer's disease. Ann Neurol. (1999) 45:358–68. 10.1002/1531-8249(199903)45:3&lt;358::AID-ANA12&gt;3.0.CO;2-X 10072051

[4] Kaye JA Swihart T Howieson D Dame A Moore MM Karnos T. Volume loss of the hippocampus and temporal lobe in healthy elderly persons destined to develop dementia. Neurology (1997) 48:1297–304. 9153461 10.1212/wnl.48.5.1297

[5] Frisoni GB Jack CR Jr. Bocchetta M Bauer C Frederiksen KS Liu Y. The EADC-ADNI Harmonized Protocol for manual hippocampal segmentation on magnetic resonance: evidence of validity. Alzheimers Dement (2015) 11:111–25. 10.1016/j.jalz.2014.05.1756 25267715 PMC4422168

[6] Mesulam MM Mufson EJ Levey AI Wainer BH. Cholinergic innervation of cortex by the basal forebrain: cytochemistry and cortical connections of the septal area, diagonal band nuclei, nucleus basalis (substantia innominata), and hypothalamus in the rhesus monkey. J Comp Neurol. (1983) 214:170–97. 10.1002/cne.902140206 6841683

[7] Bracco L Bessi V Padiglioni S Marini S Pepeu G. Do cholinesterase inhibitors act primarily on attention deficit? A naturalistic study in Alzheimer's disease patients. J Alzheimers Dis. (2014) 40:737–42. 10.3233/JAD-131154 24577458

[8] Mesulam M Shaw P Mash D Weintraub S. Cholinergic nucleus basalis tauopathy emerges early in the aging-MCI-AD continuum. Ann Neurol. (2004) 55:815–28. 10.1002/ana.20100 15174015

[9] Sassin I Schultz C Thal DR Rub U Arai K Braak E. Evolution of Alzheimer's disease-related cytoskeletal changes in the basal nucleus of Meynert. Acta Neuropathol. (2000) 100:259–69. 10.1007/s004019900178 10965795

[10] Mesulam MM. Cholinergic circuitry of the human nucleus basalis and its fate in Alzheimer's disease. J Comp Neurol. (2013) 521:4124–44. 10.1002/cne.23415 23852922 PMC4175400

[11] Teipel SJ Flatz WH Heinsen H Bokde AL Schoenberg SO Stockel S. Measurement of basal forebrain atrophy in Alzheimer's disease using MRI. Brain (2005) 128(Pt 11):2626–44. 10.1093/brain/awh589 16014654

[12] Zaborszky L Hoemke L Mohlberg H Schleicher A Amunts K Zilles K. Stereotaxic probabilistic maps of the magnocellular cell groups in human basal forebrain. NeuroImage (2008) 42:1127–41. 10.1016/j.neuroimage.2008.05.055 18585468 PMC2577158

[13] Kilimann I Grothe M Heinsen H Alho EJ Grinberg L Amaro E Jr. Subregional basal forebrain atrophy in Alzheimer's Disease: a multicenter study. J Alzheimers Dis. (2014) 40:687–700. 10.3233/JAD-132345 24503619 PMC4120953

[14] Teipel SJ Meindl T Grinberg L Grothe M Cantero JL Reiser MF. The cholinergic system in mild cognitive impairment and Alzheimer's disease: an *in vivo* MRI and DTI study. Human Brain Mapp. (2011) 32:1349–62. 10.1002/hbm.21111 20672311 PMC5899896

[15] Grothe M Zaborszky L Atienza M Gil-Neciga E Rodriguez-Romero R Teipel SJ. Reduction of basal forebrain cholinergic system parallels cognitive impairment in patients at high risk of developing Alzheimer's disease. Cereb Cortex (2010) 20:1685–95. 10.1093/cercor/bhp232 19889714 PMC2912653

[16] Muth K Schonmeyer R Matura S Haenschel C Schroder J Pantel J. Mild cognitive impairment in the elderly is associated with volume loss of the cholinergic basal forebrain region. Biol Psychiatry (2010) 67:588–91. 10.1016/j.biopsych.2009.02.026 19375072

[17] Teipel S Heinsen H Amaro E Jr. Grinberg LT Krause B Grothe M. Cholinergic basal forebrain atrophy predicts amyloid burden in Alzheimer's disease. Neurobiol Aging (2013) 35:482–91. 10.1016/j.neurobiolaging.2013.09.029 24176625 PMC4120959

[18] Hanyu H Asano T Sakurai H Tanaka Y Takasaki M Abe K. MR analysis of the substantia innominata in normal aging, Alzheimer disease, and other types of dementia. Am J Neuroradiol. (2002) 23:27–32. 11827872 PMC7975505

[19] Tanaka Y Hanyu H Sakurai H Takasaki M Abe K. Atrophy of the substantia innominata on magnetic resonance imaging predicts response to donepezil treatment in Alzheimer's disease patients. Dement Geriatr Cogn Disord. (2003) 16:119–25. 10.1159/000070998 12826736

[20] Jack CR Jr. Petersen RC Xu YC O'Brien PC Smith GE Ivnik RJ. Prediction of AD with MRI-based hippocampal volume in mild cognitive impairment. Neurology (1999) 52:1397–403. 10227624 10.1212/wnl.52.7.1397 PMC2730146

[21] Erten-Lyons D Howieson D Moore MM Quinn J Sexton G Silbert L. Brain volume loss in MCI predicts dementia. Neurology (2006) 66:233–5. 10.1212/01.wnl.0000194213.50222.1a 16434660

[22] Apostolova LG Dutton RA Dinov ID Hayashi KM Toga AW Cummings JL. Conversion of mild cognitive impairment to Alzheimer disease predicted by hippocampal atrophy maps. Arch Neurol (2006) 63:693–9. 10.1001/archneur.63.5.693 16682538

[23] Macdonald KE Bartlett JW Leung KK Ourselin S Barnes J investigators A. The value of hippocampal and temporal horn volumes and rates of change in predicting future conversion to AD. Alzheimer Disease Assoc Disord. (2013) 27:168–73. 10.1097/WAD.0b013e318260a79a 22760170 PMC4154837

[24] Ewers M Walsh C Trojanowski JQ Shaw LM Petersen RC Jack CR Jr. Prediction of conversion from mild cognitive impairment to Alzheimer's disease dementia based upon biomarkers and neuropsychological test performance. Neurobiol Aging (2012) 33:1203–14. 10.1016/j.neurobiolaging.2010.10.019 21159408 PMC3328615

[25] Csernansky JG Wang L Miller JP Galvin JE Morris JC. Neuroanatomical predictors of response to donepezil therapy in patients with dementia. Arch Neurol. (2005) 62:1718–22. 10.1001/archneur.62.11.1718 16286546

[26] Teipel SJ Cavedo E Grothe MJ Lista S Galluzzi S Colliot O. Predictors of cognitive decline and treatment response in a clinical trial on suspected prodromal Alzheimer's disease. Neuropharmacology (2016) 108:128–35. 10.1016/j.neuropharm.2016.02.005 26876309

[27] Wessels AM Dowsett SA Sims JR. Detecting Treatment Group Differences in Alzheimer's disease clinical trials: a comparison of alzheimer's disease assessment scale - Cognitive Subscale (ADAS-Cog) and the Clinical Dementia Rating - Sum of Boxes (CDR-SB). J Prev Alzheimers Dis. (2018) 5:15–20. 10.14283/jpad.2018.2 29405227 PMC12280810

[28] Sevigny JJ Peng Y Liu L Lines CR. Item analysis of ADAS-Cog: effect of baseline cognitive impairment in a clinical AD trial. Am J Alzheimers Dis Other Demen. (2010) 25:119–24. 10.1177/1533317509350298 19949163 PMC10845355

[29] Wouters H van Gool WA Schmand B Zwinderman AH Lindeboom R. Three sides of the same coin: measuring global cognitive impairment with the MMSE, ADAS-cog and CAMCOG. Int J Geriatr Psychiatry (2010) 25:770–9. 10.1002/gps.2402 19946861

[30] Birks JS Grimley Evans J. Rivastigmine for Alzheimer's disease. Cochrane Database Syst Rev. (2015) 4:CD001191. 10.1002/14651858.CD001191.pub3 25858345

[31] Loy C Schneider L. Galantamine for Alzheimer's disease. Cochrane Database Syst Rev. (2004) 4:CD001747. 10.1002/14651858.CD001747.pub2 15495017

[32] Crane PK Carle A Gibbons LE Insel P Mackin RS Gross A. Development and assessment of a composite score for memory in the Alzheimer's Disease Neuroimaging Initiative (ADNI). Brain Imaging Behav. (2012) 6:502–16. 10.1007/s11682-012-9186-z 22782295 PMC3806057

[33] Gibbons LE Carle AC Mackin RS Harvey D Mukherjee S Insel P. A composite score for executive functioning, validated in Alzheimer's Disease Neuroimaging Initiative (ADNI) participants with baseline mild cognitive impairment. Brain Imaging Behav. (2012) 6:517–27. 10.1007/s11682-012-9176-1 22644789 PMC3684181

[34] Jack CR Jr. Bernstein MA Fox NC Thompson P Alexander G Harvey D. The Alzheimer's Disease Neuroimaging Initiative (ADNI): MRI methods. J Magn Reson Imaging (2008) 27:685–91. 10.1002/jmri.21049 18302232 PMC2544629

[35] Teipel SJ Flatz W Ackl N Grothe M Kilimann I Bokde AL. Brain atrophy in primary progressive aphasia involves the cholinergic basal forebrain and Ayala's nucleus. Psychiatry Res. (2014) 221:187–94. 10.1016/j.pscychresns.2013.10.003 24434193 PMC4086659

[36] Grothe MJ Schuster C Bauer F Heinsen H Prudlo J Teipel SJ. Atrophy of the cholinergic basal forebrain in dementia with Lewy bodies and Alzheimer's disease dementia. J Neurol. (2014) 261:1939–48. 10.1007/s00415-014-7439-z 25059393

[37] Grothe M Heinsen H Teipel S. Longitudinal measures of cholinergic forebrain atrophy in the transition from healthy aging to Alzheimer's disease. Neurobiol Aging (2013) 34:1210–20. 10.1016/j.neurobiolaging.2012.10.018 23158764 PMC4058576

[38] Ashburner J. A fast diffeomorphic image registration algorithm. NeuroImage (2007) 38:95–113. 10.1016/j.neuroimage.2007.07.007 17761438

[39] Pruessner JC Li LM Serles W Pruessner M Collins DL Kabani N. Volumetry of hippocampus and amygdala with high-resolution MRI and three-dimensional analysis software: minimizing the discrepancies between laboratories. Cerebral Cortex (2000) 10:433–42. 10.1093/cercor/10.4.433 10769253

[40] Grothe MJ Ewers M Krause B Heinsen H Teipel SJ. Basal forebrain atrophy and cortical amyloid deposition in nondemented elderly subjects. Alzheimers Dement (2014) 10(5 Suppl.):S344–53. 10.1016/j.jalz.2013.09.011 24418052 PMC4092050

[41] Grothe MJ Heinsen H Amaro E Jr. Grinberg LT Teipel SJ Alzheimer's disease neuroimaging i. cognitive correlates of basal forebrain atrophy and associated cortical hypometabolism in mild cognitive impairment. Cereb Cortex (2016) 26:2411–26. 10.1093/cercor/bhv062 25840425 PMC4869802

[42] Sakamoto Y Ishiguro M Kitagawa G. Akaike Information Criterion Statistics. Boston: D. Reidel Publishing Company (1986).

[43] Ohnishi T Sakiyama Y Okuri Y Kimura Y Sugiyama N Saito T. The prediction of response to galantamine treatment in patients with mild to moderate Alzheimer's disease. Curr Alzheimer Res. (2014) 11:110–8. 10.2174/15672050113106660167 24156269 PMC3979115

[44] Efron B Tibshirani RJ. An Introduction to the Bootstrap. New York, NY: Chapman & Hall/CRC (1994). Available online at: https://www.crcpress.com/An-Introduction-to-the-Bootstrap/Efron-Tibshirani/p/book/9780412042317

[45] Raghavan N Samtani MN Farnum M Yang E Novak G Grundman M. The ADAS-Cog revisited: novel composite scales based on ADAS-Cog to improve efficiency in MCI and early AD trials. Alzheimers Dement (2013) 9(1 Suppl):S21–31. 10.1016/j.jalz.2012.05.2187 23127469 PMC3732822

[46] Van Der Putt R Dineen C Janes D Series H McShane R. Effectiveness of acetylcholinesterase inhibitors: diagnosis and severity as predictors of response in routine practice. Int J Geriatr Psychiatry (2006) 21:755–60. 10.1002/gps.1557 16906631

[47] Birks J Grimley Evans J Iakovidou V Tsolaki M Holt FE. Rivastigmine for Alzheimer's disease. Cochrane Database Syst Rev. (2009) 2:CD001191. 10.1002/14651858.CD001191.pub2 19370562

[48] Birks J Harvey RJ. Donepezil for dementia due to Alzheimer's disease. Cochrane Database Syst Rev. (2006) 1:CD001190. 10.1002/14651858.CD001190.pub2 16437430

[49] Kasper E Brueggen K Grothe MJ Bruno D Pomara N Unterauer E. Neuronal correlates of serial position performance in amnestic mild cognitive impairment. Neuropsychology (2016) 30:906–14. 10.1037/neu0000287 27182709

[50] Colliot O Chetelat G Chupin M Desgranges B Magnin B Benali H. Discrimination between Alzheimer disease, mild cognitive impairment, and normal aging by using automated segmentation of the hippocampus. Radiology (2008) 248:194–201. 10.1148/radiol.2481070876 18458242

[51] Pomara N Nolan K Halpern G. Scopolamine-induced impairment as a potential predictor of Alzheimer's disease in individuals with Apolipoprotein E type 4 alleles. Neurochem Res. (1995) 20:1519–20. 10.1007/BF00970602 8789616

[52] Snyder PJ Lim YY Schindler R Ott BR Salloway S Daiello L. Microdosing of scopolamine as a “cognitive stress test”: rationale and test of a very low dose in an at-risk cohort of older adults. Alzheimers Dement (2014) 10:262–7. 10.1016/j.jalz.2014.01.009 24698030

[53] Dumas JA Newhouse PA. The cholinergic hypothesis of cognitive aging revisited again: cholinergic functional compensation. Pharmacol Biochem Behav. (2011) 99:254–61. 10.1016/j.pbb.2011.02.022 21382398 PMC3114182

[54] Bottini G Berlingeri M Basilico S Passoni S Danelli L Colombo N. GOOD or BAD responder? Behavioural and neuroanatomical markers of clinical response to donepezil in dementia. Behav Neurol. (2012) 25:61–72. 10.1155/2012/538542 22530263 PMC5294239

[55] Mielke MM Okonkwo OC Oishi K Mori S Tighe S Miller MI. Fornix integrity and hippocampal volume predict memory decline and progression to Alzheimer's disease. Alzheimers Dement (2012) 8:105–13. 10.1016/j.jalz.2011.05.2416 22404852 PMC3305232

[56] Miettinen PS Jauhiainen AM Tarkka IM Pihlajamaki M Grohn H Niskanen E. Long-Term Response to Cholinesterase Inhibitor Treatment Is Related to Functional MRI Response in Alzheimer's Disease. Dement Geriat Cogn Disord. (2015) 40:243–55. 10.1159/000435948 26305064

[57] Zaborszky L Csordas A Mosca K Kim J Gielow MR Vadasz C. Neurons in the basal forebrain project to the cortex in a complex topographic organization that reflects corticocortical connectivity patterns: an experimental study based on retrograde tracing and 3D reconstruction. Cereb Cortex (2015) 25:118–37. 10.1093/cercor/bht210 23964066 PMC4259277

[58] Ghashghaei HT Barbas H. Neural interaction between the basal forebrain and functionally distinct prefrontal cortices in the rhesus monkey. Neuroscience (2001) 103:593–614. 10.1016/S0306-4522(00)00585-6 11274781

